# Desmin is not ubiquitously expressed in head and neck muscle fibers

**DOI:** 10.1111/joa.70240

**Published:** 2026-09-18

**Authors:** Per Stål, Jingxia Liu, Fatima Pedrosa Domellöf, Farhan Shah

**Affiliations:** ^1^ Department of Medical and Translational Biology Umeå University Umeå Sweden; ^2^ Department of Clinical Science, Ophthalmology Umeå University Umeå Sweden

**Keywords:** craniofacial muscles, cytoskeletal proteins, Desmin, muscle, myosin heavy chain

## Abstract

Desmin, a major muscle‐specific intermediate filament protein, is widely regarded as a ubiquitous component of mature skeletal muscle fibers. However, recent observations in human extraocular and palatal muscles indicate that a subset of fibers lack or show very weak desmin expression. The aim of the study was to investigate the presence of desmin in 21 different adult, two toddler, and one fetal human head and neck muscles. The samples were analyzed by immunohistochemistry, and, in one muscle group, also by in situ hybridization for desmin mRNA. Two limb muscles, the biceps brachii and the vastus lateralis, served as controls. In limb muscles, all fibers consistently exhibited desmin immunoreactivity. In contrast, fibers with absent or very weak desmin immunoreactivity were identified across all examined head and neck muscles, with substantial variation between muscle groups. These fibers were predominantly associated with the slow MyHC1 phenotype. A similar pattern was also observed in infant and fetal samples. In longitudinal sections, some fibers showed segmental absence of desmin along their length. In situ hybridization demonstrated absent or markedly reduced desmin mRNA in fibers with absent or reduced desmin immunoreactivity. Taken together, this study provides the first evidence that subsets of myofibers in most human head and neck muscles lack desmin immunoreactivity, indicating that desmin is not ubiquitous in all myofibers in these muscles. These findings reveal previously unrecognized cytoskeletal heterogeneity within human head and neck musculature. The functional significance of fibers lacking or showing very weak desmin immunoreactivity remains to be determined.

## Graphical abstract

Desmin is not ubiquitously expressed in human head and neck muscle fibers. A subset of fibers showed absent or very weak desmin immunoreactivity, with corresponding reduction or absence of desmin mRNA expression.

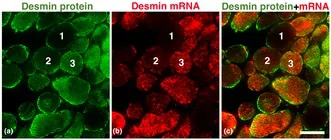

## 1 INTRODUCTION

We have previously demonstrated that certain craniofacial muscles exhibit intricate morphologies and a more complex composition of contractile proteins than limb muscles (Shah et al., 2016, Stål et al., 1994, Yu et al., 2002, Janbaz et al., 2014, Kjellgren et al., 2003). In adult humans, limb muscles typically contain three myosin heavy chain (MyHC) isoforms, the major motor proteins that determine contraction velocity and force. In contrast, jaw muscles express six MyHC isoforms, whereas extraocular muscles (EOMs) can contain up to eight different MyHC isoforms (Schiaffino et al., 2015, Korfage & van Eijden, 1999, Stål et al., 1994, Kjellgren et al., 2003). In addition to this diversity in contractile proteins, we have reported that a subgroup of fibers in the human soft palate and EOMs differs from limb muscles by lacking, or partly lacking, immunohistochemical expression of the cytoskeletal protein desmin in both healthy and diseased states (Liu & Pedrosa Domellöf, 2020; Shah et al., 2016; Shah, Franklin, et al., 2019; Shah, Forsgren, et al., 2019; Shah & Stål, 2022). More recently, we showed that the muscle fibers in the soft palate lacking desmin protein expression also lack detectable desmin mRNA, suggesting that this feature reflects a distinct molecular phenotype (Stål et al., 2024). This is particularly interesting because desmin has been regarded as a ubiquitous and essential component of mature human muscle, with its absence primarily associated with genetic muscle disorders (Clemen et al., 2013).

Desmin is the most abundant intermediate filament (IF) protein in muscle fibers, forming a three‐dimensional cytoskeletal scaffold that extends throughout the entire muscle fiber. Structurally, desmin links adjacent myofibrils at the Z‐discs and anchors the most peripheral myofibrils to the sarcolemma, nuclear membrane, and intracellular organelles such as mitochondria. Beyond its structural function in stabilizing and transmitting force, cytoskeletal proteins are involved in multiple cellular processes, including maintenance of cell shape, signal transduction, growth, division, differentiation, and intracellular organelle transport (Capetanaki et al., 2007). More recent studies indicate that desmin filaments may also regulate proteostasis and cell size (Agnetti et al., 2022). In skeletal muscle, dynamic remodeling of desmin filaments has been proposed to facilitate catabolic processes as an adaptive response to changing physiological or environmental conditions (Agnetti et al., 2022; Cohen et al., 2012; Volodin et al., 2017). Furthermore, post‐translational modifications and misfolding of desmin, particularly in cardiac muscle, have been identified as important factors influencing cellular homeostasis and disease progression (Agnetti et al., 2022). Given desmin's central role in maintaining muscle fiber integrity (Capetanaki et al., 2007; Paulin & Li, 2004), the discovery of muscle fibers lacking desmin in human muscles represents a significant paradigm shift (Liu & Pedrosa Domellöf, 2020; Shah et al., 2016; Shah, Franklin, et al., 2019; Shah, Forsgren, et al., 2019; Shah & Stål, 2022). At present, it is unclear whether desmin‐deficient muscle fibers are prevalent in the head and neck region. Therefore, the objective of this study was to investigate whether the absence of desmin immunoreactivity is a common characteristic of head and neck muscles. To achieve this, we analyzed the presence of the intermediate‐filament protein desmin in 21 different human head and neck muscles and in two limb muscles.

## 2 MATERIALS AND METHODS

### 2.1 Muscle samples

A total of 40 muscle samples from 21 different head and neck muscles were studied. The samples were obtained post‐mortem from 13 previously healthy donors (10 males, 3 females, mean age 54 years, age range 26–81 years) who died suddenly from accidental causes and by biopsy from one subject (palate muscle, male, 49 years). In addition, samples were also collected at autopsy from the palatopharyngeus muscle of two male infants (age 10 days and 1.4 years) and one extraocular (EOM) muscle from a fetus at 18 weeks of gestation. For comparison, three muscle samples from the arm, specifically the biceps brachii muscle (2 females, 1 male, mean age 49 years, range 18–98 years), and two samples from the leg, the vastus lateralis muscle (2 males, mean age 27 years, 23 and 30 years, respectively), were obtained post‐mortem. One additional vastus lateralis muscle sample was obtained by biopsy technique (male, 20 years). All subjects were previously healthy, and their medical histories did not reveal any known significant diseases or medications. The muscles and number of samples are shown in Table 1.

**TABLE 1:** Antibody specificity and dilution.

Antibody	Product code	Specificity	Gene^a^	Host/clone	Dilution	Source
Desmin	M0760	Human and animal desmin	DES	mAb‐mouse/D33	1: 100	1
Desmin	ab15200	Human and animal desmin	DES	pAb rabbit	1:200	2
Laminin	Z0097	Human and animal laminin	LAM	pAb rabbit	1:500	1
MyHC	A4.840	MyHC1	MYH7	mAb‐mouse	1:300	3
MyHC	BA‐D5	MyHC1	MYH7	mAb‐mouse	1:500	3
MyHC	A4.74	MyHC2a	MYH7	mAb‐mouse	1:500	3

*Note*: 1. Dako, Sweden, 2. Abcam, UK; 3. Developmental Studies Hybridoma Bank, developed under the auspices of the NICHD and maintained by the University of Biological Sciences, Iowa City, Iowa, USA. Gift from Stefano Schiaffino, CNR Inst. of Neuroscience, Padova, Italy.

^a^ Official gene nomenclature according to OMIM (http://www.ncbi.nlm.nih.gov/omim/).

The samples were carefully oriented and either immediately mounted in OCT compound (Tissue Tek, Miles, Elkhart, IN, USA) and frozen in liquid propane chilled with liquid nitrogen, or fixed before freezing using 4% formaldehyde in 0.1 M phosphate buffer, pH 7.0, for 24 h at 4**°**C and overnight washing at 4**°**C in Tyrodes solution containing 10% sucrose. The specimens were then stored at −80°C until further use. Both fixed and unfixed samples were included in the immunohistochemical analyses.

### 2.2 Immunohistochemistry

Immunohistochemical staining was performed using modified standard techniques and previously well‐characterized monoclonal (mAb) and polyclonal (pAb) antibodies (Abs). For details of the antibodies and dilutions, see Table 1. Desmin expression in muscle was detected using a highly specific monoclonal antibody (mAb M0760, clone D33) that shows no cross‐reactivity with other intermediate filament proteins. A polyclonal antibody (pAb 15,200) against desmin was used to reconfirm its expression in muscle fibers. Both antibodies detect the expected ~53 kDa desmin protein on Western blots. A polyclonal antibody directed against laminin (pAb Z0097) was used to visualize the basal lamina of the muscle cell membranes. For identification of MyHC isoforms in the fibers, two mAbs (A4.840 and BA‐D5) against the slow MyHC1 and one mAb (A4.74) against fast MyHC2A were used.

The muscle specimens were cut in a cryostat at −20°C into 5 μm‐thick serial cross‐sections and mounted on Superfrost Plus Adhesion Slides (Epredia GmbH, Germany). The sections were immersed in 5% normal non‐immune donkey serum (Jackson ImmunoResearch Laboratories, West Grove, PA, USA) for 15 min and rinsed in 0.01 M phosphate‐buffered saline (PBS) for 3 × 5 min. Afterward, the sections were incubated in a humidified chamber with the primary antibody diluted to the appropriate concentration in PBS containing bovine serum albumin. Double‐staining procedures were performed by adding an anti‐laminin pAb. Incubation was carried out overnight at 4°C. After additional washes in PBS, sections were incubated with the secondary Ab (37°C for 30 min) and washed in PBS for 3 × 5 min. Bound primary Abs were visualized by indirect immunofluorescence using affinity‐purified Abs prepared for multiple labelling and conjugated with fluorochrome with different emission spectra; fluorescein (FITC), Rhodamine Red‐X (RRX; Jackson ImmunoResearch Laboratories, West Grove, PA, USA), and Alexa Fluor 488 (Invitrogen, CA, USA). The sections were then mounted in Vectashield Mounting Medium (H‐1000) or in Mounting Medium with 4′,6‐diamidino‐2‐phenylindole (DAPI) for nuclear staining (Vector Laboratories, Burlingame, CA, USA). Control sections were treated as above, except that the primary Abs were exchanged with non‐immune serum. For details of the multi‐immunostaining technique, see (Lindstrom & Thornell, 2009).

### 2.3 In situ hybridization

In situ hybridization was performed on muscle cross‐sections by using Riboprobe targeting desmin mRNA (BC032116, BioScience UK) according to a protocol previously described (Schaeren‐Wiemers & Gerfin‐Moser, 1993). Unfixed, snap‐frozen muscle samples were used. Due to the susceptibility of RNA to post‐mortem degradation, in situ hybridization analyses were limited to freshly obtained palate muscle biopsies with optimal tissue preservation. For mRNA visualization, Red TR/Naphthol S‐MX and TRIS tablets (SIGMA‐ALDRICH Co, Spruce Street, St Louis, USA) were used. Immediately after in situ hybridization, immunohistochemical staining with polyclonal antibody (pAb) (15200) against desmin (DES) was performed on the same section. For details, see (Stål et al., 2024).

### 2.4 Muscle fiber phenotype classification

Based on the immunostaining pattern for the different MyHC mAbs, the muscle fibers were either classified as slow fibers containing MyHC1 or fast‐contracting fibers containing MyHC2, regardless of co‐expression of other special MyHC isoforms reported in cranial muscles. Hybrid fibers co‐expressing MyHC1 and MyHC2 were sorted into either the MyHC1 or MyHC2 group based on the antibody showing the strongest reaction.

### 2.5 Analysis and quantification

In each muscle cross‐section double‐stained with desmin mAb M0760 and pAb laminin Z0097, two to four random areas were scanned at 20 × magnification under a fluorescence microscope (Leica DM6000B; Leica Microsystems CMS GmbH, Wetzlar, Germany) equipped with a digital high‐speed fluorescence CCD camera (Leica DFC360 FX). Based on the staining pattern for desmin, muscle fibers showing clear desmin immunoreactivity were classified as desmin‐positive fibers, whereas fibers lacking or showing very weak desmin immunoreactivity, including staining confined to the fiber periphery, were classified as desmin‐reduced/negative fibers. The various immunostaining patterns for desmin and their relation to MyHC1 or MyHC2 proteins in muscle fibers were quantified manually in each photo (Photoshop CS5, version 12.0.4, San Jose, CA, USA). The investigators were blinded to the origin of the samples. A total of 44,906 muscle fibers were analyzed.

### 2.6 Ethical approval

The regional Medical Ethical Committee in Umeå approved the study (5254‐177/84, 95‐275, 05‐130 M). Autopsy specimens were collected in agreement with Swedish Laws and regulations on autopsy and transplantation. All material was anonymized and not traceable to any individual. The samples were collected in agreement with the Declaration of Helsinki.

## 3 RESULTS

### 3.1 Antibody reaction pattern for desmin

**FIGURE 1:**
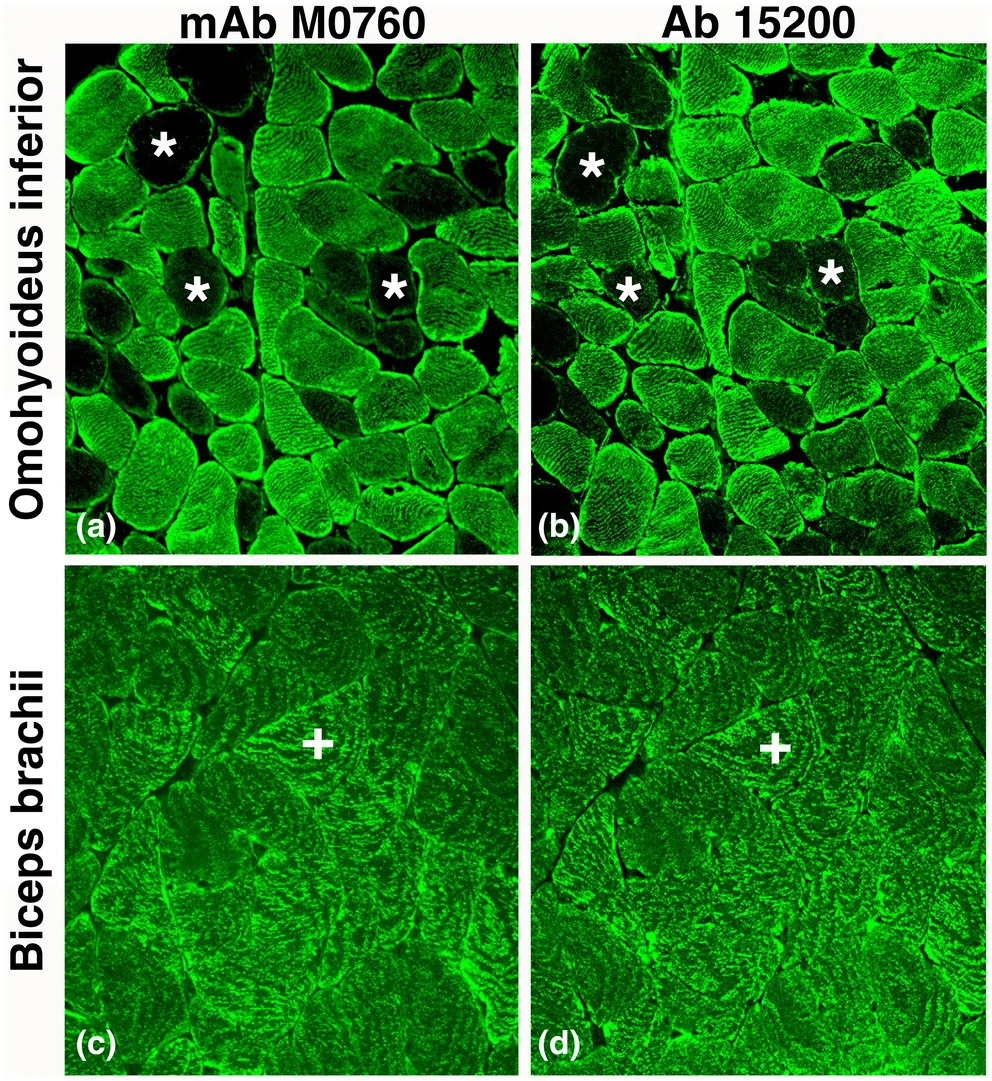
Serial cross‐sections of a neck muscle, musculus omohyoideus inferior (a, b), and a limb muscle, musculus biceps brachii (c, d), immunostained with a monoclonal (M0760) and a polyclonal (15200) antibody against desmin. Note the lack or very weak desmin immunoreactivity, including staining confined to the fiber periphery in some fibers (*) in the neck muscle, as shown by both antibodies. In the limb muscle, both desmin antibodies labeled all fibers. The signs in a and b (*) and in c and d (+) mark the same fiber in serial sections. Scale bar 50 μm.

**FIGURE 2:**
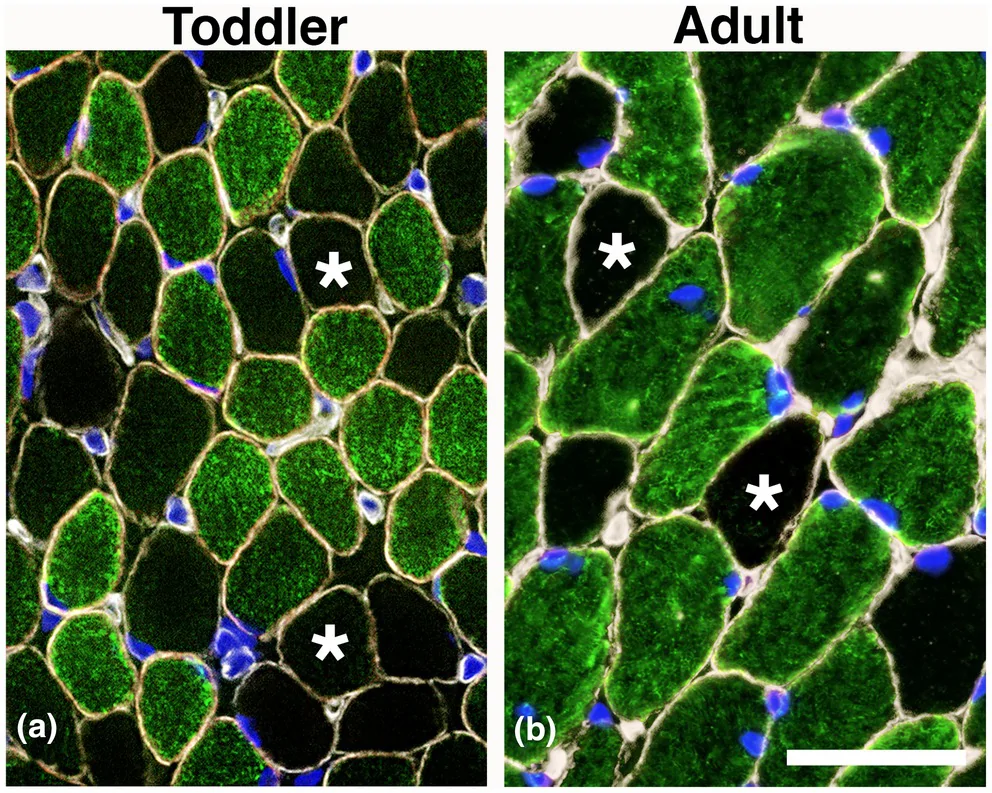
Muscle cross‐sections of the palatopharyngeus muscle stained for desmin (green), laminin (white), and DAPI (blue nuclei) from a 16‐month‐old toddler (a) and an adult (b). Note that muscle fibers lacking or showing very weak desmin immunoreactivity, including staining confined to the fiber periphery (*) are present in both toddlers and adults. Scale bar 50 μm.

**FIGURE 3:**
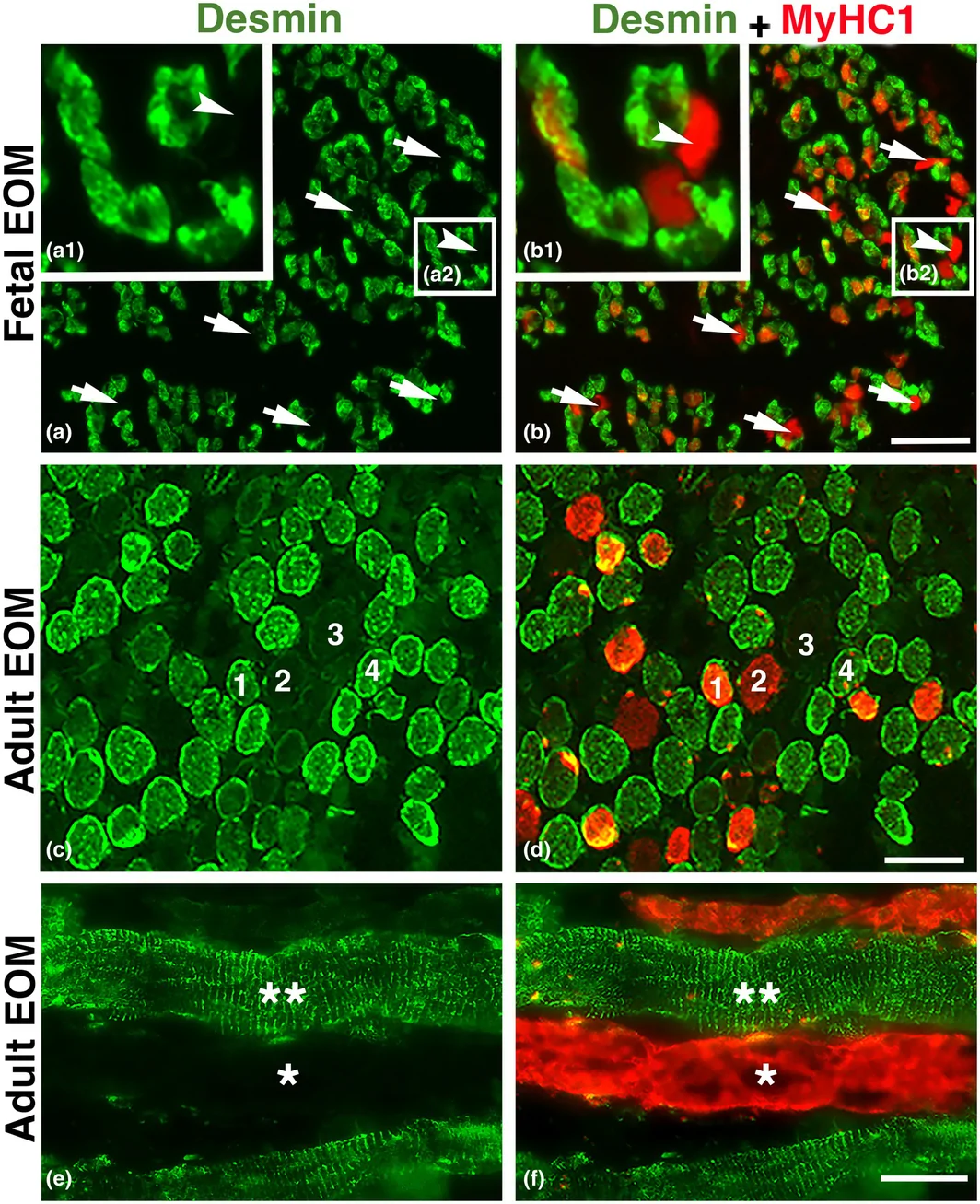
Muscle cross‐sections (a–d) and longitudinal sections (e, f) of an extraocular muscle from an 18‐week fetus (a, b), and an adult (c–f) donor stained for desmin (green) and MyHC1 (red). Arrows in a indicate muscle fibers lacking or showing very weak desmin immunoreactivity, and arrows in b show the corresponding fibers double‐stained for desmin and MyHC1. Insets a1 and b1 in figures a and b show magnifications of the corresponding areas marked a2 and b2, respectively. Longitudinally sectioned fibers (e, f) showing lack of or weak desmin immunoreactivity in fibers expressing MyHC1 are marked (*). Fibers with immunoreactivity for desmin but not MyHC1 are marked (**). Some longitudinally sectioned fibers exhibited desmin immunoreactivity that appeared restricted to part of the fiber length (e, f). Scale bars 50 μm.

**FIGURE 4:**
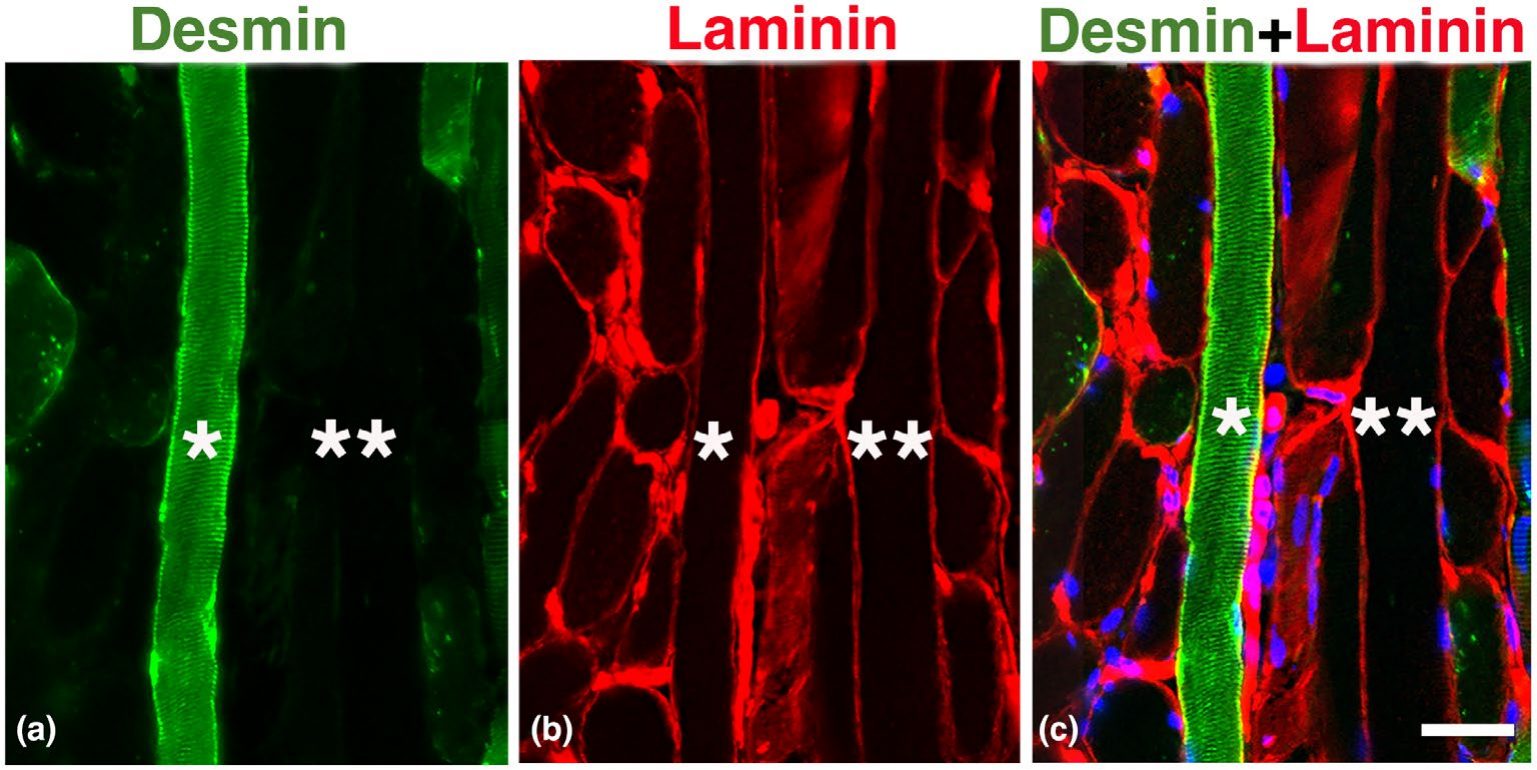
Longitudinal section of the uvula muscle immunostained for desmin (green, a) and laminin (red, b). Figure c shows a merged image of desmin and laminin. A fiber with immunoreactivity for desmin is marked (*), and a fiber lacking desmin expression throughout its length is marked (**). Scale bar 50 μm.

**FIGURE 5:**
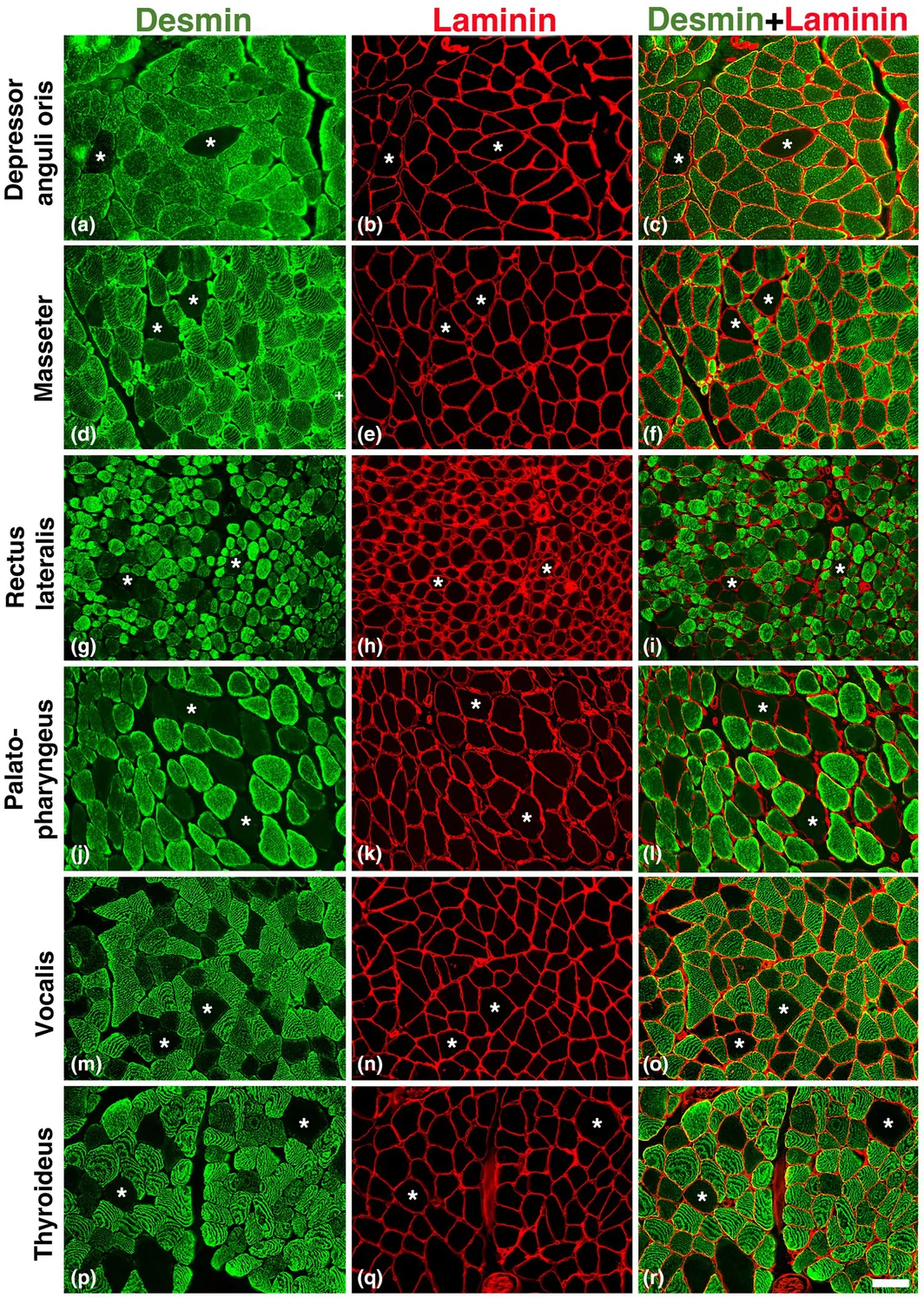
Cross‐sections of different head and neck muscles immunostained for desmin (green, left panel), laminin (red, middle panel), and merged staining (right panel). The panels display a selected muscle from the facial (a–c), jaw (d–f), extraocular (g–i), soft palate (j–l), laryngeal (m–o), and infrahyoidal muscle groups (p‐r). Muscle fibers lacking or showing very weak desmin immunoreactivity, including staining confined to the fiber periphery are marked (*). Scale bar 50 μm.

**FIGURE 6:**
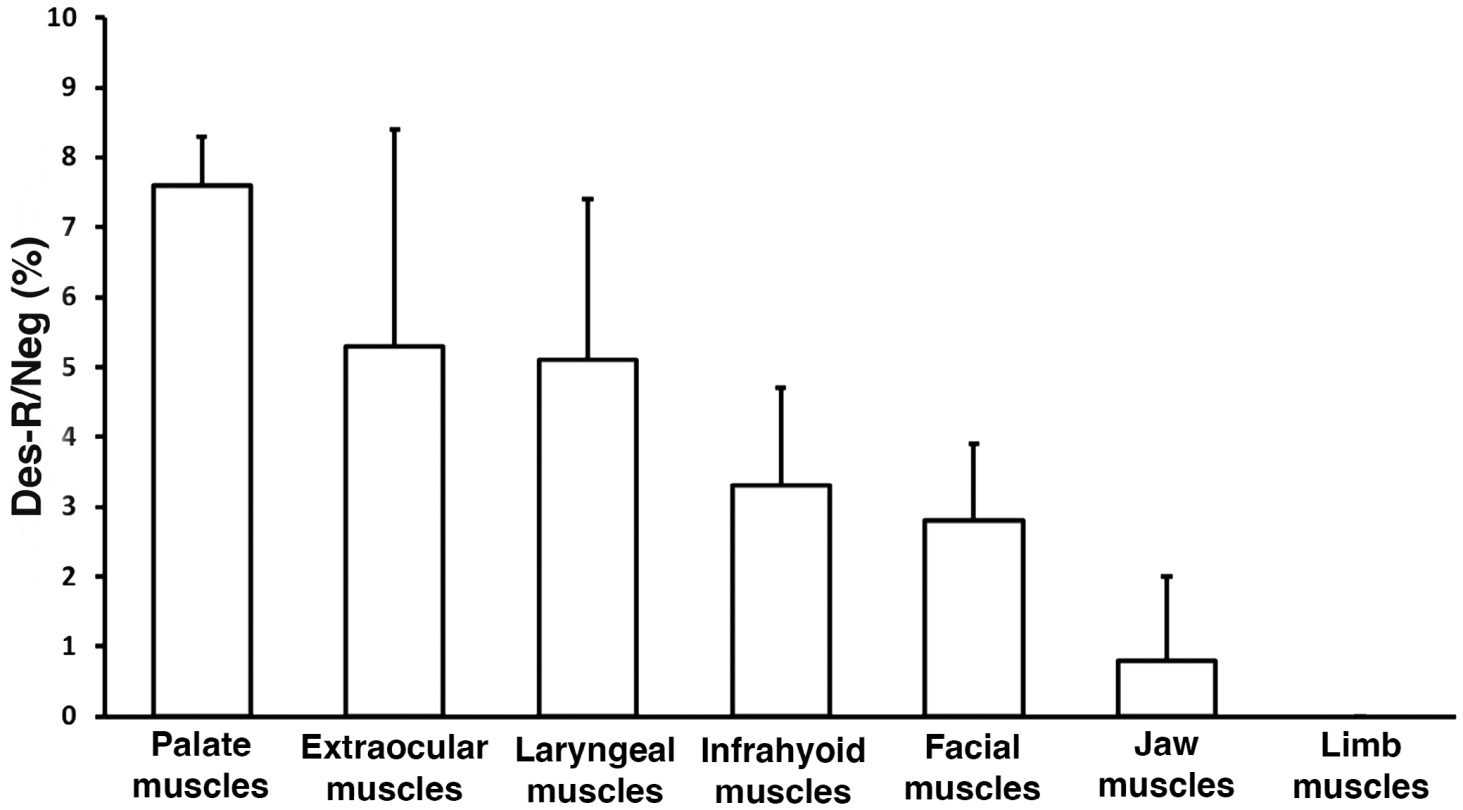
Bar graphs showing the frequency of fibers lacking or showing very weak or reduced desmin immunoreactivity, including staining confined to the fiber periphery (Des‐R/Neg) in the head, neck, and limb muscle groups.

Immunolabeling with the monoclonal (M0760) and polyclonal (15200) antibodies against desmin showed similar staining patterns in autopsy and biopsy samples, as well as in fixed and unfixed tissue. The staining pattern of these antibodies in a neck and a limb muscle is shown in Figure 1. All sections in which the primary Abs were replaced with non‐immune serum were unstained. In cross‐sections of biceps brachii and vastus lateralis muscles, all fibers showed moderate to strong immunoreactivity to Abs against desmin (Figures 1, 2, 3, 4, 5, 6), and no fibers were identified that lacked detectable staining (Figure 1). In contrast, a subset of muscle fibers in the head and neck muscles showed very weak desmin immunoreactivity in adults (Figures 5 and 6). Similar patterns were observed in the palatopharyngeus muscle of toddlers and in the EOM of an 18‐week fetus (Figures 2 and 3). In longitudinal sections, some fibers exhibited immunostaining only in specific regions along their length (Figures 3 and 4).

### 3.2 Frequency of muscle fibers expressing different MyHC isoforms

**FIGURE 7:**
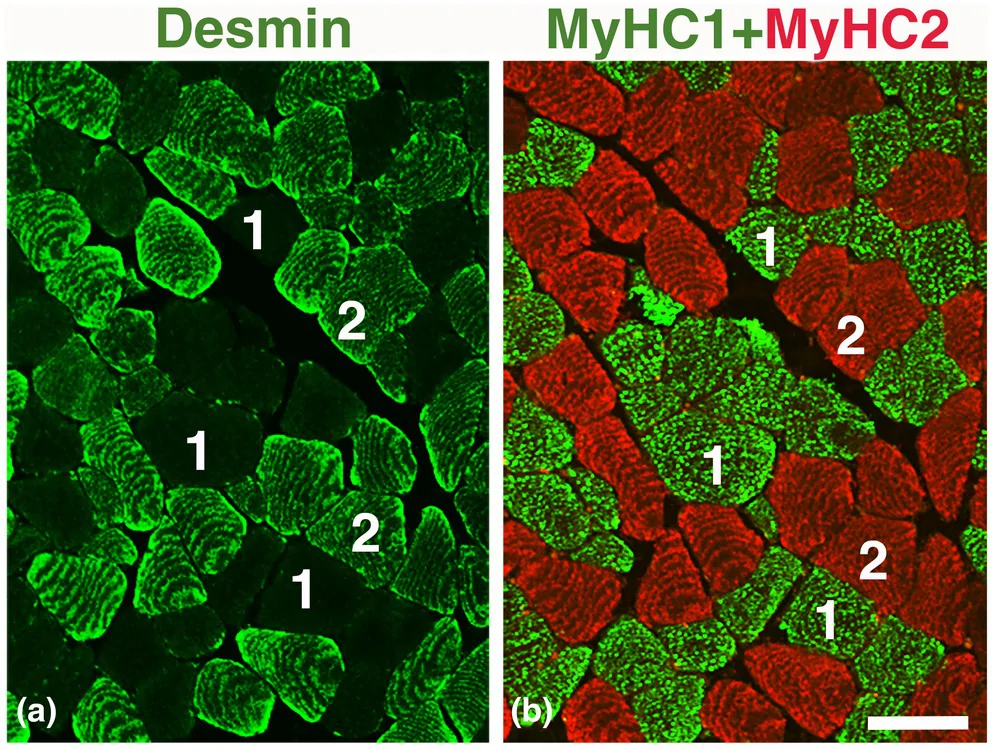
Serial cross‐sections of the musculus vocalis immunostained for desmin (a, green) and double‐stained for MyHC1 (green) and MyHC2 (red) in panel b. Muscle fibers expressing MyHC1 are marked 1, and those expressing MyHC2 are marked 2. Note that muscle fibers lacking or showing very weak desmin immunoreactivity preferentially express MyHC1. Scale bar 50 μm.

All muscle groups, except the jaw muscles, predominantly contained fibers expressing fast MyHC2 isoforms (Figure 7). The highest percentages of MyHC2 fibers were found in the extraocular and palate muscles (75.0 ± 6.5% and 73.0 ± 2.8%, respectively), whereas the anterior masseter muscle had the highest percentage of fibers expressing MyHC1 (68 ± 5%). The frequencies of fiber phenotypes in each muscle are shown in Table 2.

**TABLE 2:** Frequency (%) of fibers lacking or showing very weak desmin immunoreactivity in 21 head and neck and two limb muscles, and their distribution in muscle fibers expressing MyHC1 or MyHC2.

Muscles	Number	Des‐pos	Des‐neg	Des‐neg in MyHC fiber phenotypes		Proportion of MyHC fiber phenotypes		Embryological origin
				MyHC1	MyHC2	MyHC1	MyHC2	
		% SD	% SD	% SD	% SD	% SD	% SD	Mesodermal components of:
Facial muscles								
Zygomaticus major	3	95.7 ± 1.4	4.3 ± 1.4	4.2 ± 1.3	0.1 ± 0.1	22 ± 4	78 ± 4	Second pharyngeal arch
Zygomaticus minor	2	98.5 ± 0.2	1.5 ± 0.2	1.3 ± 0.03	0.2 ± 0.3	13 ± 6	87 ± 6	Second pharyngeal arch
Buccinator	1	97.4	2.6	2.6	0	53	47	First pharyngeal arch
Depressor anguli oris	1	97.8	2.2	2.2	0	41	59	Second pharyngeal arch
Depressor labii inferior	1	96.4	3.6	3.6	0	39	61	Second pharyngeal arch
Mean ± SD		**97 ± 1.2**	**2.8 ± 1.1**	**2.8 ± 1.1**	**0.1 ± 0.1**	**33.6 ± 15.9**	**66.4 ± 15.9**	
Jaw muscles								
Anterior masseter	2	99.9 ± 0.1	0.1 ± 0.1	0.1 ± 0.1	0	68 ± 5	32 ± 5	First pharyngeal arch
Posterior masseter	2	97.9 ± 1.1	2.1 ± 1.3	2.1 ± 1.3	0	54 ± 10	46 ± 10	First pharyngeal arch
Temporalis	2	99.9 ± 0.1	0.1 ± 0.1	0.1 ± 0.1	0	43 ± 5	57 ± 5	First pharyngeal arch
Mean ± SD		**99.2 ± 1.2**	**0.8 ± 1.2**	**0.8 ± 1.2**	**0.0**	**55.0 ± 12.5**	**45.0 ± 12.5**	
Extraocular muscles								
Rectus medialis	3	93.5 ± 0.8	6.5 ± 0.8	5.7 ± 1.1	0.8 ± 0.2	19 ± 2	81 ± 2	Somites^a^
Rectus superior	2	92.5 ± 3.6	7.6 ± 3.6	5.9 ± 2.8	1.7 ± 0.8	24 ± 1	76 ± 1	Somites^a^
Rectus lateralis	2	98.2 ± 0.1	1.8 ± 0.1	1.7 ± 0.2	0.1 ± 0.1	32 ± 15	68 ± 15	Somites^a^
Mean ± SD		**94.7 ± 3.0**	**5.3 ± 3.1**	**4.4 ± 2.3**	**0.9 ± 0.8**	**25.0 ± 6.5**	**75.0 ± 6.5**	
Palate muscles								
Uvula	3	92.9 ± 3.7	7.1 ± 3.7	5.4 ± 3.3	2 ± 1	25 ± 8	75 ± 8	Second pharyngeal arch
Palatopharyngeus	3	91.9 ± 1.4	8.1 ± 1.3	6.0 ± 1.1	2 ± 2	29 ± 6	71 ± 6	Fourth pharyngeal arch
Mean ± SD		**92.4 ± 0.7**	**7.6 ± 0.7**	**5.7 ± 0.4**	**2.0 ± 0.0**	**27.0 ± 2.8**	**73.0 ± 2.8**	
Laryngeal muscles								
Cricothyroideus	3	95.1 ± 1.9	4.9 ± 1.9	4.0 ± 1.6	0.9 ± 0.8	38 ± 7	62 ± 7	Fourth and sixth pharyngeal arches
Vocalis	1	97	3	3	0	51	49	Fourth and sixth pharyngeal arches
Thyroepiglottus	1	91.5	8.5	8.5	0	46	54	Fourth and sixth pharyngeal arches
Mean ± SD		**94.3 ± 3.1**	**5.1 ± 2.3**	**4.6 ± 2.5**	**0.4 ± 0.5**	**46.1 ± 5.8**	**53.8 ± 5.8**	
Infrahyoid muscles								
Omohyoideus superior	3	98.3 ± 0.7	1.7 ± 0.7	1.4 ± 0.8	0.3 ± 0.1	30 ± 9.1	70 ± 9.1	First pharyngeal arch
Omohyoideus inferior	1	94.7	5.3	2.4	2.9	38	62	Second pharyngeal arch
Sternohyoideus	1	97.5	2.5	2.5	0	36	64	Second and third pharyngeal arches
Sternothyroideus	1	96.8	3.2	3.2	0	37	63	Second and third pharyngeal arches
Thyrohyoideus	2	96.0 ± 0.9	4.0 ± 0.9	3.2 ± 0.9	0.8 ± 0.1	50 ± 2	50 ± 2	Third and fourth pharyngeal arches
Mean ± SD		**96.7 ± 1.5**	**3.3 ± 1.4**	**2.5 ± 0.7**	**0.8 ± 1.2**	**38.1 ± 7.1**	**61.9 ± 7.1**	
Limb muscles								
Biceps brachii	3	100	0	‐	‐	46 ± 6	54 ± 6	Somites
Vastus lateralis	3	100	0	‐	‐	41 ± 3	59 ± 3	Somites
Mean ± SD		**100 ± 0**	0	‐	‐	**43.5 ± 4.5**	**56.5 ± 4.5**	

*Note*: The overall proportion of fibers expressing MyHC1 or MyHC2 isoforms in the different muscles is given. Values are present as mean and **±** SD. Bold values represent the calculated proportions of fibers with reduced or absent desmin immunoreactivity and are presented as descriptive data (mean ± SD). No statistical significance testing was performed for these values, as no fibers with reduced or absent desmin immunoreactivity were observed in the limb muscle controls.

^a^ Prechordal and cranial paraxial mesoderm.

### 3.3 Frequency of desmin‐negative muscle fibers in head and neck muscles

Desmin‐negative fibers were observed in varying proportions across all analyzed head and neck muscles (Table 1, Figure 6). The highest proportions of desmin‐negative fibers were observed in the palate and EOMs (7.6 ± 0.7% and 5.3 ± 3.1%, respectively), whereas only a few desmin‐negative fibers were observed in the jaw muscles (0.8 ± 1.2%). Across all cranial muscles, 84% of desmin‐negative muscle fibers were MyHC1 fiber phenotypes (Figure 7). The frequency of desmin‐negative fibers in muscles and across different fiber phenotypes is shown in Table 1.

### 3.4 Head and neck muscles

In the facial muscles, the mean proportion of desmin‐negative fibers was 2.8 ± 1.1%. The highest proportion was observed in the zygomaticus major muscle (4.3 ± 1.4%), and the lowest in the zygomaticus minor muscle (1.5 ± 0.2%). In the buccinator, depressor anguli oris, and depressor labii inferioris, all desmin‐negative fibers were exclusively of the slow MyHC1 type. In contrast, the zygomaticus major and minor muscles contained, in addition to desmin‐negative MyHC1 fibers, a small proportion of desmin‐negative myofibers expressing fast MyHC2 isoforms, see Figure 5.

**FIGURE 8:**
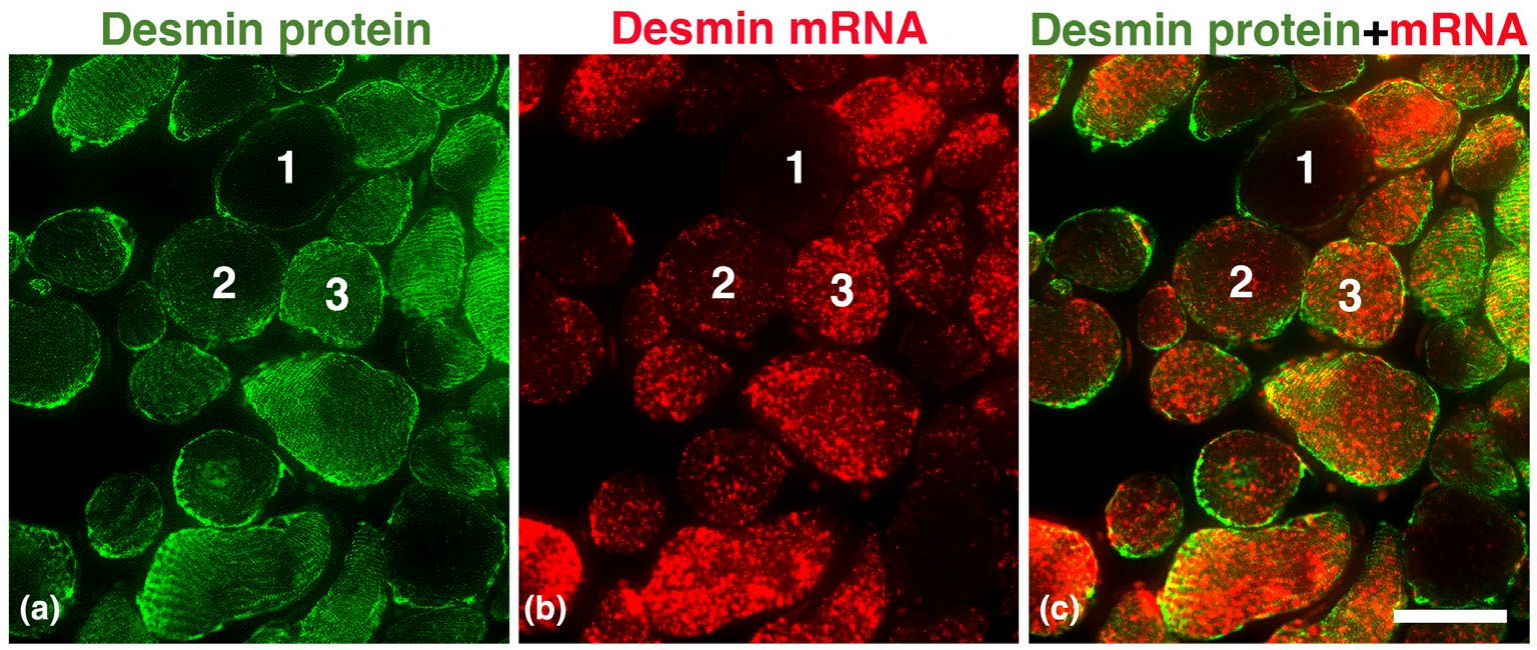
Cross‐sections of the uvula muscle immunostained for desmin protein (green, a) and hybridized with a riboprobe against desmin mRNA (red, b). Merged staining for desmin protein and desmin mRNA is shown in c. Muscle fibers lacking or showing very weak desmin immunoreactivity, including staining confined to the fiber periphery and its mRNA are marked 1. Fibers displaying low (1), moderate (2), or high (3) mRNA expression in desmin‐positive fibers are marked. Scale bar 50 μm.

All jaw muscle samples (Figure 5) examined showed a very low proportion of desmin‐negative muscle fibers, averaging 0.8 ± 1.2%. The posterior part of the masseter muscle, which was dominated by MyHC1 fibers, had the highest proportion of desmin‐negative fibers (2.1 ± 1.3%). All desmin‐negative fibers in the jaw muscles were of the slow MyHC1 phenotype.

In EOMs (Figure 3), the mean proportion of desmin‐negative muscle fibers in cross‐sections was 5.3 ± 3.1%, with substantial variation across subjects and muscle samples. The rectus superior muscles had the highest proportion of desmin‐negative fibers (7.6 ± 3.6%). Most desmin‐negative myofibers in EOMs contained MyHC1.

The palate muscle group (Figures 4 and 5) had the highest mean proportion of desmin‐negative fibers (7.6 ± 0.7%) among all head and neck muscles analyzed. The palatopharyngeus muscle had the highest proportion of desmin‐negative fibers (8.1 ± 1.3%). Both the uvula and the palatopharyngeus muscles contained desmin‐negative fibers across both MyHC1 and MyHC2 fiber phenotypes.

The mean proportion of desmin‐negative muscle fibers in laryngeal muscles was 5.1 ± 2.3%. The thyroepiglottic muscle had the highest proportion of desmin‐negative muscle fibers (8.5%), whereas the vocalis muscles (Figure 5) displayed the lowest (3.0%). In these samples, all desmin‐negative myofibers were of the slow MyHC1 phenotype.

The infrahyoid muscle group had a mean proportion of 3.3 ± 1.4% desmin‐negative fibers, with the highest frequency in the inferior omohyoid muscle (Figure 1) (5.3%) and the lowest in the sternohyoid muscle (2.5%). All desmin‐negative fibers in the sternohyoideus and thyreoideus muscles (Figure 5) were of the MyHC1 type; the remaining muscles in this group had desmin‐negative fibers in both MyHC1 and MyHC2 fiber phenotypes.

### 3.5 Desmin mRNA expression in relation to desmin protein

In situ hybridization was performed on palate muscle biopsies to assess desmin mRNA expression in relation to desmin protein distribution. Muscle fibers with normal desmin immunoreactivity showed a relatively homogeneous distribution of desmin mRNA throughout the cytoplasm. In contrast, fibers lacking or showing reduced desmin protein displayed absent or markedly reduced desmin mRNA signal (Figure 8). In some fibers with segmental absence of desmin protein, the corresponding regions also lacked detectable desmin mRNA.

## 4 DISCUSSION

The present study extends our previous observations by demonstrating that desmin‐negative fibers are present across a broad range of human head and neck muscles. While all fibers in limb muscles showed immunolabeling for desmin, a distinct subpopulation of fibers in the head and neck muscles exhibited markedly reduced or absent desmin immunoreactivity. Fibers with absent or very weak desmin immunoreactivity were observed at varying frequencies across the head and neck muscles examined, with the highest mean frequencies in the palate, extraocular, and laryngeal muscle groups and the lowest in the jaw muscles. Our previous and current observations demonstrate that desmin‐negative fibers also lack desmin mRNA, supporting the interpretation that this feature reflects a distinct cytoskeletal phenotype rather than a technical or post‐translational effect (Stål et al., 2024). These findings are particularly noteworthy given that desmin has long been regarded as a ubiquitous and indispensable component of skeletal muscle, essential for maintaining myofibrillar alignment and structural integrity (Capetanaki et al., 2007; Paulin & Li, 2004). Thus, our findings challenge the prevailing paradigm of desmin ubiquity in human skeletal muscle and reveal greater heterogeneity in desmin expression among muscle fibers of the human head and neck than previously recognized. (Paulin & Li, 2004).

Each muscle in the human body is specialized to generate force and produce movements adapted to specific functional demands. Although much of the existing data on human skeletal muscle has focused on limb and trunk muscles, increasing attention in recent decades has been directed toward the head and neck musculature, expanding our understanding of muscle diversity in the human body (Stål et al., 1994, Stål et al., 1995, Yu et al., 2002, Shah et al., 2016, Stål et al., 1990, Stål et al., 1987, Stål et al., 2024, Liu & Pedrosa Domellöf, 2020, Janbaz et al., 2014).

Many head and neck muscles display structural and functional features that distinguish them from limb muscles. These differences are highlighted by the unique and more complex fiber type composition in these muscles (Stål et al., 1994, Stål et al., 1995, Yu et al., 2002, Shah et al., 2016, Stål et al., 1990, Stål et al., 1987, Kjellgren et al., 2003). Unlike most limb muscles, orofacial and EOMs are small and lack attachment to bone at one or both ends. Moreover, many of these muscles are arranged in multiple orientations and interact from different directions, so the contraction of one muscle can impose oblique or transverse forces on adjacent muscle bellies. A slightly different functional arrangement is observed in the infrahyoid muscles, primarily contributing to positioning and stabilizing the hyoid‐laryngeal complex. Consequently, these muscles do not generate joint movements and are designed to produce rapid, relatively low‐force contractions. It is considered that desmin has an important role in transmitting lateral forces to the extracellular matrix (ECM) and neighboring myofibrils during muscle contraction. However, many craniofacial muscles, including the palate and EOMs, have loosely packed muscle fibers abundantly surrounded by connective tissue, indicating limited interfiber connectivity (Stål et al., 1987, Stål & Lindman, 2000, Mcloon et al., 2018). Moreover, as many of these muscles are arranged in multiple orientations and interact from different directions, so the contraction of one muscle may impose oblique or transverse forces on adjacent muscle bellies. In contrast, human jaw muscles, which had the lowest proportion of fibers lacking or showing very weak desmin immunoreactivity, more closely resemble typical limb muscles in that they are relatively larger, attach to bone at both ends, and span a joint. Taken together, the anatomical and physiological differences underscore that the musculature of the head and neck cannot be regarded as merely analogous to limb musculature. Instead, these muscles constitute a distinct group compared with limb muscles in terms of their anatomy, function, and developmental origin. These findings reveal previously unrecognized heterogeneity in desmin expression within human head and neck musculature.

Desmin is one of the earliest muscle‐specific proteins expressed during embryonic myogenesis and undergoes further structural maturation as skeletal muscle develops (Li et al., 1993). The presence of desmin‐negative fibers in the EOM of a fetus and in the palatopharyngeus muscle of toddlers indicates that this phenotype is established early in development rather than acquired later in life. These findings suggest that the absence of desmin immunoreactivity in subsets of muscle fibers represents a normal developmental phenotype in human head and neck muscles.

Additional evidence for an atypical heterogeneity in desmin expression in a subset of head and neck muscle fibers comes from studies showing that fibers lacking desmin immunoreactivity also exhibit reduced or absent immunoreactivity for the C‐terminal domain of dystrophin (Shah, Franklin, et al., 2019). Dystrophin is a key structural protein that links the muscle fiber cytoskeleton to the surrounding extracellular matrix (ECM) via the sarcolemma. Its C‐terminal domain associates with the membrane‐spanning dystrophin‐associated protein complex (DAPC), while the N‐terminal domain binds actin filaments, collectively maintaining sarcolemmal stability and facilitating the transmission of sarcomeric force to the ECM (Campbell, 1995; Petrof et al., 1993; Srivastava & Yu, 2006). These observations suggest that the molecular coupling between cytoskeletal and membrane‐associated protein complexes differs across a population of palate muscle fibers (Shah et al., 2016). Previous studies from our group showed that desmin‐negative fibers in the human EOMs and soft palate did not exhibit vimentin immunoreactivity, indicating that vimentin does not compensate for the absence of desmin in these fibers (Janbaz et al., 2014; Liu & Pedrosa Domellöf, 2020; Shah et al., 2016). Whether similar expression patterns of other cytoskeletal proteins occur in additional head and neck muscles or other specialized muscle groups remains unknown and warrants further investigation. Collectively, these findings indicate that desmin‐negative fibers are associated with a distinct cytoskeletal protein organization rather than a simple substitution of desmin by other intermediate filament proteins. The functional significance of this phenotype remains to be determined.

Reduced or absent desmin immunoreactivity is classically associated with pathogenic variants in DES and other genes causing desmin‐related myopathies (Clemen et al., 2013). Although genetic analyses were beyond the scope of the present study, none of the individuals included had a known history or clinical features suggestive of hereditary muscle disease. Moreover, the desmin‐negative phenotype has been consistently observed in multiple independent cohorts, including healthy individuals, in our previous studies (Shah et al., 2016; Shah, Franklin, et al., 2019; Shah & Stål, 2022; Stål et al., 2024). Future studies integrating histological and genetic analyses will be important to determine whether genetic variation contributes to this phenotype in any subset of individuals.

A notable finding was that in longitudinally sectioned muscle samples, the absence of desmin in some fibers was confined to segmental regions along the fiber length, suggesting heterogeneity in desmin expression among muscle fibers. Although we did not systematically examine desmin expression along entire fibers in all muscles, we observed, consistent with previous reports, a segmental pattern with absence of desmin in several fibers of the palate and EOMs (Janbaz et al., 2014; Shah et al., 2016). Moreover, we have previously reported that desmin is absent or reduced in the vicinity of single en plaque neuromuscular junctions (NMJs) in a large proportion of EOM myofibers, as well as in MyHC slow tonic/MyHC1 fibers displaying multiple en grappe endings (Liu & Pedrosa Domellöf, 2020).

In situ hybridization analyses further support that the absence of desmin protein in a subset of muscle fibers is a true biological phenomenon. In biopsy samples, fibers lacking desmin immunoreactivity also showed absent or markedly reduced desmin mRNA expression, whereas fibers with normal desmin protein expression displayed a relatively homogeneous distribution of desmin transcripts. In some fibers with segmental absence of desmin protein, the corresponding regions also lacked detectable desmin mRNA. A similar correspondence between desmin protein and mRNA expression has previously been reported, including segmental patterns along individual fibers (Stål et al., 2024). Although post‐translational modifications may influence antibody immunoreactivity and mRNA abundance does not always correlate directly with protein expression (Gygi et al., 1999), the consistent reduction of desmin immunoreactivity and DES mRNA in the same muscle fibers argues against post‐translational modifications alone as the sole explanation for the observed staining pattern. Future studies using antibody‐independent proteomic approaches may help further validate the heterogeneous distribution of desmin and characterize the molecular composition of desmin‐negative muscle fibers. A limitation of this study was that mRNA analyses were restricted to biopsy samples from a single muscle group, as RNA analysis is suboptimal in autopsy specimens.

In the present study, desmin‐negative fibers were predominantly observed among MyHC1‐expressing fibers across all head and neck muscles examined. A similar association between desmin deficiency and expression of slow‐tonic MyHC in EOMs has been reported previously (Stål et al., 2024). Although a lack of desmin was occasionally observed in fast‐contracting MyHC2 fibers, it appears to be primarily associated with MyHC1 fibers exhibiting sustained, slow, low‐force activity. Interestingly, the highest number of desmin‐negative fibers was found in muscles with a relatively small proportion of slow‐twitch MyHC1 fibers, whereas the lowest proportion of desmin‐negative fibers was observed in the jaw muscles, which have a high proportion of MyHC1 fibers.

The observed molecular and structural differences from limb muscles may be associated with their distinct embryonic origins. While limb muscles develop from somites, many craniofacial, pharyngeal, laryngeal, and EOMs arise from mesoderm associated with the pharyngeal arches or the prechordal mesoderm, often in close interaction with neural crest–derived connective tissues (Noden & Francis‐West, 2006). These differences in developmental origin are known to shape gene regulatory networks and protein expression profiles. Distinct developmental programs may therefore contribute to the unique cytoskeletal phenotypes observed in craniofacial muscles. Future studies are needed to determine whether alternative desmin isoforms or other cytoskeletal components contribute to the distinct heterogeneity in desmin expression among muscle fibers, and whether similar fibers occur in other muscle groups throughout the human body.

In conclusion, desmin expression is heterogeneous among muscle fibers in the human head and neck region, with a distinct subgroup of desmin‐negative fibers present in orofacial and neck muscles. The consistent absence of desmin immunoreactivity in some muscle fibers, supported by a corresponding lack of desmin mRNA, indicates a distinct cytoskeletal organization. This distinct cytoskeletal architecture highlights the molecular heterogeneity of head and neck muscles and warrants further functional investigation.

## AUTHOR CONTRIBUTIONS

Per Stål and Farhan Shah were responsible for the conceptualization and primary funding of the study. Per Stål, Farhan Shah, Jingxia Liu, and Fatima Pedrosa Domellöf collected biopsies, performed immunostaining, and analyses. All authors contributed to the evaluation of data, writing, and editing of the manuscript. Farhan Shah and Per Stål were responsible for the project administration and final editing. All authors have read and approved the final manuscript.

## FUNDING INFORMATION

Open access funding provided by Umea University. This work was supported by the Swedish Research Council (Dnr 2018‐02574, Per Stål) and Kempestiftelserna (JCSMK23‐0001 and JCSMK25‐0083, Farhan Shah).

## Data Availability

The data that support the findings of this study are available on request from the corresponding author. The data are not publicly available due to privacy or ethical restrictions.
